# Advantages of the nested case-control design in diagnostic research

**DOI:** 10.1186/1471-2288-8-48

**Published:** 2008-07-21

**Authors:** Cornelis J Biesheuvel, Yvonne Vergouwe, Ruud Oudega, Arno W Hoes, Diederick E Grobbee, Karel GM Moons

**Affiliations:** 1 Julius Center for Health Sciences and Primary Care, University Medical Center, Utrecht, The Netherlands; 2 The Children's Hospital at Westmead, Sydney, Australia

## Abstract

**Background:**

Despite its benefits, it is uncommon to apply the nested case-control design in diagnostic research. We aim to show advantages of this design for diagnostic accuracy studies.

**Methods:**

We used data from a full cross-sectional diagnostic study comprising a cohort of 1295 consecutive patients who were selected on their suspicion of having deep vein thrombosis (DVT). We draw nested case-control samples from the full study population with case:control ratios of 1:1, 1:2, 1:3 and 1:4 (per ratio 100 samples were taken). We calculated diagnostic accuracy estimates for two tests that are used to detect DVT in clinical practice.

**Results:**

Estimates of diagnostic accuracy in the nested case-control samples were very similar to those in the full study population. For example, for each case:control ratio, the positive predictive value of the D-dimer test was 0.30 in the full study population and 0.30 in the nested case-control samples (median of the 100 samples). As expected, variability of the estimates decreased with increasing sample size.

**Conclusion:**

Our findings support the view that the nested case-control study is a valid and efficient design for diagnostic studies and should also be (re)appraised in current guidelines on diagnostic accuracy research.

## Background

In diagnostic research it is essential to determine the accuracy of a test to evaluate its value for medical practice [1]. Diagnostic test accuracy is assessed by comparing the results of the index test with the results of the reference standard in the same patients. Given the cross-sectional nature of a diagnostic accuracy question, the design may be referred to as a cross-sectional cohort design. The (cohort) characteristic by which the study subjects (cohort members) are selected is 'the suspicion of the target disease', defined by the presence of particular symptoms or signs [2]. The collected study data allow for calculation of all diagnostic accuracy parameters of the index test, such as sensitivity, specificity, odds ratio, receiver operating characteristic (ROC) curve and predictive values, i.e. the probabilities of presence and absence of the disease given the index test result(s).

Subjects are not always selected on their initial suspicion of having the disease but often on the true presence or absence of the disease among those who underwent the reference test in routine care practice, which merely reflects a cross-sectional case-control design [3,4]. Appraisal of such conventional case-control design in diagnostic accuracy research has been limited due to its problems related to the incorrect sampling of cases and controls [3-7]. These problems may be overcome by applying a *nested *(cross-sectional) case-control study design, which may be advantageous over a full (cross-sectional) cohort design. The rationale, strengths and limitations of a nested case-control approach in epidemiology studies have widely been discussed in the literature [8-11], but not so much in the context of diagnostic accuracy research [6].

We therefore aim to show advantages of the nested case-control design for addressing diagnostic accuracy questions and discuss its pros and cons in relation to a conventional case-control design and to the full (cross sectional) cohort design in this domain. We will illustrate this with data from a recently conducted diagnostic accuracy study.

### Case-control versus nested case-control design

The essence of a case-control study is that cases with the condition under study arise in a source population and controls are a representative sample of this same source population. Not the entire population is studied, what would be a full cohort study or *census *approach, but rather a *random sample *from the source population [12]. A major flaw inherent to case-control studies, described as early as 1959 [13], is the difficulty to ensure that cases and controls are a representative sample of the same source population. In a nested case-control study the cases emerge from a well-defined source population and the controls are sampled from that same population. The main difference between a case-control and a nested case-control study is that in the former the cases and controls are sampled from a source population with unknown size, whereas the latter is 'nested' in an existing predefined source population with known sample size. This source population can be a group or cohort of subjects that is followed over time or not.

The term 'cohort' is commonly referred to a group of subjects followed over time in etiologic or prognostic research. But in essence, time is no prerequisite for the definition of a cohort. A cohort is a group of subjects that is defined by the same characteristic. This characteristic can be a particular birth year, a particular living area, and also the presence of a particular sign or symptom that makes them suspected of having a particular disease as in diagnostic research. Accordingly, a cross-sectional study can either be a cross-sectional case-control study or a cross-sectional cohort study.

### Case-control and nested case-control design in diagnostic accuracy research

In diagnostic accuracy research the case-control design is incorrectly applied when subjects are selected from routine care databases. First, this design commonly leads to biased estimates of diagnostic accuracy of the index test due to referral or (partial) verification bias [4,14-18]. In routine care, physicians selectively refer patients for additional tests, including the reference test, based on previous test results. This is good clinical practice but a bad starting point for diagnostic research. As said, for diagnostic research purposes all subjects suspected of the target disease preferably undergo the index test(s) plus reference test irrespective of previous test results. Second, selection of patients with a negative reference test result as 'controls' may lead to inclusion of controls that correspond to a different clinical domain, i.e. patients who underwent the reference test but not necessarily because they were similarly suspected of the target condition [16,17]. A third disadvantage of such case-control design is that absolute probabilities of disease presence given the index test results, i.e. the predictive values or post-test probabilities, that are the desired parameters for patient care, cannot be obtained. Cases and controls are sampled from a source population of unknown size. The total number of patients that were initially suspected of the target disease based on the presence of symptoms or signs, i.e. the true source population, is commonly unknown as in routine care patients are hardly classified by their symptoms and signs at presentation [18]. Hence, the sampling fraction of cases and controls is unknown and valid estimates of the absolute probabilities of disease presence cannot be calculated [12].

**Figure 1:**
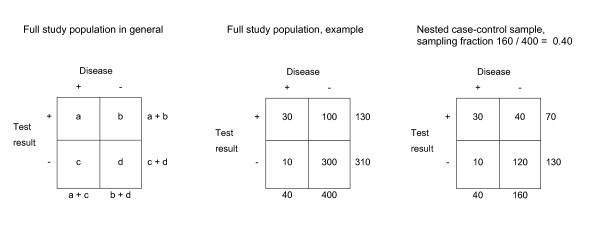
**Theoretical example of a full study population and a nested case-control sample**. The index test result and the outcome are obtained for all patients of the study population. The case-control ratio was 1:4 (sampling fraction (SF) = 160/400 = 0.40). Valid diagnostic accuracy measures can be obtained from the nested case-control sample, by multiplying the controls with 1/sampling fraction. For example, the positive predictive value (PPV) of a full study population can be calculated with a/(a + b), in this example 30/(30 + 100) = 0.23. In a nested case-control sample the PPV is calculated with a/(a + (1/SF)*b), in this example: 30/(30 + 2.5*40) = 0.23. In a case-control sample however, the controls are sampled from a source population with unknown size. Therefore, the sample fraction is unknown and valid estimate of the PPV cannot be calculated.

A nested case-control study in diagnostic research includes the full population or cohort of patients suspected of the target disease. The 'true' disease status is obtained for all these patients with the reference standard. Hence, there is no referral or partial verification bias. The results of the index tests can then be obtained for all subjects with the target condition but only for a sample of the subjects without the target condition. Usually all patients with the target disease are included, but this could as well be a sample of the cases. Besides the absence of bias, all measures of diagnostic accuracy, including the positive and negative predictive values, can simply be obtained by weighing the controls with the case-control sampling fraction, as explained in Figure 1.

### Potential advantages of a nested case-control design in diagnostic research

The nested case-control study design can be advantageous over a full cross-sectional cohort design when actual disease prevalence in subjects suspected of a target condition is low, the index test is costly to perform, or if the index test is invasive and may lead to side effects. Under these conditions, one limits patient burden and saves time and money as the index test is performed in only a sample of the control subjects.

Furthermore, the nested case-control design is of particular value when stored data (serum, images etc.) of an existing study population are re-analysed for diagnostic research purposes. Using a nested case-control design, only data of a sample of the full study population need to be retrieved and analysed without having to perform a new diagnostic study from the start. This may for example apply to evaluation of tumour markers to detect cancer, but also for imaging or electrophysiology tests.

Diagnostic accuracy estimates derived from a nested case-control study, should be virtually identical to a full cohort analysis. However, the variability of the accuracy estimates will increase with decreasing sample size. We illustrate this with data of a diagnostic study on a cohort of patients who were suspected of DVT.

## Methods

### Patients

A cross-sectional study was performed among a cohort of adult patients suspected of deep vein thrombosis (DVT) in primary care. This suspicion was primarily defined by the presence of a painful and swollen or red leg that existed no longer than 30 days. Details on the setting, data collection and main results have been described previously. [19,20] In brief, the full study population included 1295 consecutive patients who visited one of the participating primary care physicians with above symptoms and signs of DVT. Patients were excluded if pulmonary embolism was suspected. The general practitioner systematically documented information on patient history and physical examination. Patient history included information such as age, gender, history of malignancy, and recent surgery. Physical examination included swelling of the affected limb and difference in circumference of the calves calculated as the circumference (in centimetres) of affected limb minus circumference of unaffected limb, further referred to as calf difference test. Subsequently, all patients were referred to undergo D-dimer testing. In line with available guidelines and previous studies, the D-dimer test result was considered abnormal if the test yielded a D-dimer level ≥ 500 ng/ml. [21,22] Finally, they all underwent the reference test, i.e. repeated compression ultrasonography (CUS) of the lower extremities. In patients with a normal first CUS measurement, the CUS was repeated after seven days. DVT was considered present if one CUS measurement was abnormal. The echographist was blinded to the results of patient history, physical examination, and the D-dimer assay.

### Nested case-control samples

Nested case-control samples were drawn from the full study population (n = 1295). In all samples, we included always all 289 cases with DVT. Controls were randomly sampled from the 1006 subjects without DVT. We applied four different and frequently used case-control ratios, i.e. one control for each case (1:1), two controls for each case (1:2), three controls for each case (1:3) and four controls for each case (1:4). For example, a sample with case-control ratio of 1:1 contained 289 cases and 289 random subjects out of 1006 controls (sampling fraction 289/1006 = 0.287). In the 1:4 approach, we sampled with replacement. For each case-control ratio, 100 nested case-control samples were drawn.

### Statistical analysis

We focussed on two important diagnostic tests for DVT, i.e. the dichotomous D-dimer test and the continuous calf difference test. The latter was specifically chosen as it allowed for the estimation and thus comparison of the area under the ROC curve (ROC area). Diagnostic accuracy measures of both tests were estimated for the four case-control ratios and compared with those obtained from the full study population. Measures of diagnostic accuracy included sensitivity and specificity, positive and negative predictive values and the odds ratio (OR) for the D-dimer test, and the OR and the ROC area for the calf difference test.

In the analysis of the nested case-control samples, we multiplied control samples by [1/sample fraction] corresponding to the case-control ratio (1:1 = 3.48; 1:2 = 1.74; 1:3 = 1.16; 1:4 = 0.87). For each case-control ratio, the point estimates and variability were determined. The median estimate of the 100 samples was considered as the point estimate. Analyses were performed using SPSS version 12.0 and S-plus version 6.0.

## Results

In the full study population, the prevalence of DVT was 22% (n = 289), the D-dimer test was abnormal in 69% of the patients (n = 892) and the mean difference in calf circumference was 2.3 cm (Table 1). The prevalence of DVT was 50%, 33%, 25% and 20% in the nested case-control samples as a result of the sampling ratios (1:1, 1:2, 1:3 and 1:4, respectively). The distributions of the test characteristics in the control samples were similar as for the patients from the full study population without DVT (Table 1).

**Table 1:** Distribution of test results in the full study population and the nested case-control samples with various case-control ratios

	Full study population		Nested case-control samples for different case-control ratios				
		
	DVT +	DVT-	Cases	Controls			
				
Test results	n = 289	n = 1006	n = 289	1:1 n = 289	1:2 n = 578	1:3 n = 867	1:4 n = 1156
D-dimer test abnormal	271 (94)	621 (62)	271 (94)	178 (61)	357 (62)	535 (62)	713 (62)
Calf difference, cm*	3.2 (1.7)	2.1 (1.6)	3.2 (1.7)	2.1 (1.6)	2.1 (1.6)	2.1 (1.6)	2.1 (1.6)
							
Age, years*	61.9 (16.8)	59.4 (17.7)	61.9 (16.8)	59.4 (17.8)	59.5 (17.8)	59.4 (17.8)	59.4 (17.7)
Male gender	137 (47)	330 (33)	137 (47)	94 (33)	190 (33)	282 (33)	380 (33)

For each case-control ratio, 100 nested case-control samples were drawn. The statistics of the control samples are the average values. All values represent absolute patient numbers (%) unless stated otherwise.

DVT+ = deep vein thrombosis present; DVT- = deep vein thrombosis absent; *mean (standard deviation)

In the full study population the sensitivity and negative predictive value were high for the D-dimer test, 0.94 and 0.96, respectively (Table 2), whereas the specificity and positive predictive value were relatively low. The OR for the calf difference test was 1.44 and the ROC area was 0.69.

**Table 2:** Estimates of diagnostic accuracy with 95% confidence intervals for the D-dimer and calf difference tests obtained in the full study population

Measures of diagnostic accuracy	D-dimer test (dichotomous)	Calf difference test (continuous per cm)
Sensitivity	0.94 (0.91 – 0.97)	-
Specificity	0.38 (0.35 – 0.41)	-
PPV	0.30 (0.27 – 0.33)	-
NPV	0.96 (0.94 – 0.98)	-
Odds Ratio	9.33 (5.70 – 15.3)	1.44 (1.33 – 1.56)
ROC area	-	0.69 (0.65 – 0.72)

- = not applicable; PPV = positive predictive value; NPV = negative predictive value ROC area = area under the receiver operating characteristic curve

**Figure 2:**
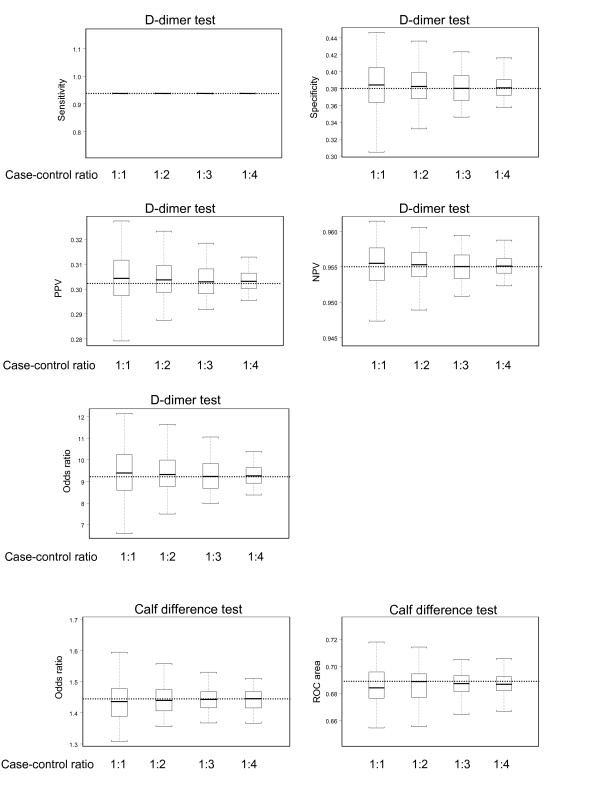
**Estimates of diagnostic accuracy of the D-dimer test and calf difference test for the 100 nested case-control samples with case-control ratios ranging from 1:1 to 1:4**. The boxes indicate mean values and corresponding interquartile ranges (25^th ^and 75^th ^percentile). Whiskers indicate 2.5^th ^and 97.5^th ^percentiles. The dotted lines represent the values estimated in the full study population.

The average estimates of diagnostic accuracy for each of the four case-control ratios were similar to the corresponding estimates of the full study population (Figure 2). For example, the negative predictive value of the D-dimer test was 0.955 in both the full study population and for the four case-control ratios. The OR of the calf difference test was 1.44 in the full study population and the OR derived from the nested case-control samples were on average also 1.44.

## Discussion

The use of (conventional) case-control studies in diagnostic research has often been associated with biased estimates of diagnostic accuracy, due to the incorrect sampling of subjects [3-6,18]. Moreover, this study design does not allow for the estimation of the desired absolute disease probabilities. We discussed and showed that a case-control study nested within a well defined cohort of subjects suspected of a particular target disease with known sample size can yield valid estimates of diagnostic accuracy of an index test, including the absolute probabilities of disease presence or absence. Diagnostic accuracy parameters derived from a full (cross-sectional) cohort of patients suspected of DVT were similar to the estimates derived from various nested case-control samples averaged over 100 simulations. Expectedly, the variability decreased with increasing number of controls, making the measures estimated in the larger case-control samples more precise.

As discussed, the number of subjects from which the index test results need to be retrieved can substantially be reduced with a nested case-control design. Hence, the nested case-control design is particularly advantageous when the prevalence of the target condition in the cohort of patients suspected of the target disease is rare, when the index test results are costly or difficult to collect and for re-analysing stored images or specimen. However, precision of the diagnostic accuracy measures will be hampered by increased variability when too little control patients are included.

Rutjes et al nicely discussed limitations of different study designs in diagnostic research [6]. They proposed the 'two-gate design with representative sampling' (which resembles the nested case-control design in this paper) as a valid design. We confirmed their proposition with a quantitative analysis of a diagnostic study. Rutjes et al suggested not to use the term 'nested case-control' to prevent confusion with etiologic studies where this design is commonly applied. Indeed, diagnostic and etiologic research differs fundamentally, first and foremost on the concept of time. Diagnostic accuracy studies are, in contrast to etiologic studies, typically cross-sectional in nature. Furthermore, diagnostic associations between index and reference tests are purely descriptive, whereas in etiologic studies causal associations and potential confounding are involved. Despite these major differences we believe there is no reason not to use the term nested case-control study in diagnostic research as well. The term inherently refers to the method of sampling of study subjects which can be the same in a diagnostic or etiologic setting, and has no direct bearing on the other issues typically related to etiologic case control studies.

## Conclusion

Our findings support the view that the nested case-control study is a valid and efficient design for diagnostic studies. We believe that the nested case-control approach should be applied more often in diagnostic research, and also be (re)appraised in current guidelines on diagnostic methodology.

## Competing interests

The authors declare that they have no competing interests.

## Authors' contributions

All authors commented on the draft and the interpretation of the findings, read and approved the final manuscript. CJB was responsible for the design, statistical analysis and wrote the original manuscript. YV was responsible for the design and statistical analysis. RO was responsible for the data collection. AWH was responsible for expertise in case-control design. DEG and KGMM were responsible for conception and design of the study and coordination.

## Pre-publication history

The pre-publication history for this paper can be accessed here:



## References

[B1] Knottnerus JA, van Weel C, Muris JW (2002). Evaluation of diagnostic procedures. BMJ.

[B2] Knottnerus JA, Muris JW (2003). Assessment of the accuracy of diagnostic tests: the cross-sectional study. J Clin Epidemiol.

[B3] Lijmer JG, Mol BW, Heisterkamp S, Bonsel GJ, Prins MH, Meulen van der JHP, Bossuyt PMM (1999). Empirical evidence of design-related bias in studies of diagnostic tests. JAMA.

[B4] Rutjes AW, Reitsma JB, Di Nisio M, Smidt N, van Rijn JC, Bossuyt PM (2006). Evidence of bias and variation in diagnostic accuracy studies. CMAJ.

[B5] Whiting P, Rutjes AW, Reitsma JB, Glas AS, Bossuyt PM, Kleijnen J (2004). Sources of variation and bias in studies of diagnostic accuracy: a systematic review. Ann Intern Med.

[B6] Rutjes AW, Reitsma JB, Vandenbroucke JP, Glas AS, Bossuyt PM (2005). Case-control and two-gate designs in diagnostic accuracy studies. Clin Chem.

[B7] Kraemer H (1992). Evaluating Medical Tests.

[B8] Mantel N (1973). Synthetic retrospective studies and related topics. Biometrics.

[B9] Essebag V, Genest J, Suissa S, Pilote L (2003). The nested case-control study in cardiology. Am Heart J.

[B10] Ernster VL (1994). Nested case-control studies. Prev Med.

[B11] Langholz B, Armitage PCT (2005). Case-Control Study, Nested. Encyclopedia of Biostatistics.

[B12] Rothman KJ, Greenland S (1998). Modern epidemiology.

[B13] Mantel N, Haenszel W (1959). Statistical aspects of the analysis of data from retrospective studies of disease. J Natl Cancer Inst.

[B14] Ransohoff DF, Feinstein AR (1978). Problems of spectrum and bias in evaluating the efficacy of diagnostic tests. N Engl J Med.

[B15] Begg CB, Greenes RA (1983). Assessment of diagnostic tests when disease verification is subject to selection bias. Biometrics.

[B16] Knottnerus JA, Leffers JP (1992). The influence of referral patterns on the characteristics of diagnostic tests. J Clin Epidemiol.

[B17] van der Schouw YT, van Dijk R, Verbeek ALM (1995). Problems in selecting the adequate patient population from existing data files for assessment studies of new diagnostic tests. J Clin Epidemiol.

[B18] Oostenbrink R, Moons KG, Bleeker SE, Moll HA, Grobbee DE (2003). Diagnostic research on routine care data: prospects and problems. J Clin Epidemiol.

[B19] Oudega R, Hoes AW, Moons KG (2005). The Wells rule does not adequately rule out deep venous thrombosis in primary care patients. Ann Intern Med.

[B20] Oudega R, Moons KG, Hoes AW (2005). Limited value of patient history and physical examination in diagnosing deep vein thrombosis in primary care. Fam Pract.

[B21] Perrier A, Desmarais S, Miron M, de Moerloose P, Lepage R, Slosman D, Didier D, Unger P, Patenaude J, Bounameaux H (1999). Non-invasive diagnosis of venous thromboembolism in outpatients. Lancet.

[B22] Schutgens RE, Ackermark P, Haas FJ, Nieuwenhuis HK, Peltenburg HG, Pijlman AH, Pruijm M, Oltmans R, Kelder JC, Biesma DH (2003). Combination of a normal D-dimer concentration and a non-high pretest clinical probability score is a safe strategy to exclude deep venous thrombosis. Circulation.

